# Hippo signaling suppresses tumor cell metastasis via a Yki-Src42A positive feedback loop

**DOI:** 10.1038/s41419-021-04423-y

**Published:** 2021-12-03

**Authors:** Yan Ding, Guiping Wang, Meixiao Zhan, Xiaohan Sun, Yanran Deng, Yunhe Zhao, Bin Liu, Qingxin Liu, Shian Wu, Zizhang Zhou

**Affiliations:** 1 State Key Laboratory of Crop Biology, College of Life Sciences, Shandong Agricultural University, 271018 Tai’an, China; 2 Tianjin Key Laboratory of Protein Sciences, State Key Laboratory of Medical Chemical Biology, College of Life Sciences, Nankai University, 300071 Tianjin, China; 3 Center of Intervention radiology, Zhuhai Precision Medicine Center, Zhuhai People’s Hospital, 519000 Zhuhai, China; 4 Jiangsu Key laboratory of Drug Screening, China Pharmaceutical University, 210009 Nanjing, China

**Keywords:** Metastasis, Epithelial-mesenchymal transition

## Abstract

Metastasis is an important cause of death from malignant tumors. It is of great significance to explore the molecular mechanism of metastasis for the development of anti-cancer drugs. Here, we find that the Hippo pathway hampers tumor cell metastasis in vivo. Silence of *hpo* or its downstream *wts* promotes tumor cell migration in a Yki-dependent manner. Furthermore, we identify that inhibition of the Hippo pathway promotes tumor cell migration through transcriptional activating *src42A*, a *Drosophila* homolog of the *SRC* oncogene. Yki activates *src42A* transcription through direct binding its intron region. Intriguingly, Src42A further increases Yki transcriptional activity to form a positive feedback loop. Finally, we show that *SRC* is also a target of YAP and important for YAP to promote the migration of human hepatocellular carcinoma cells. Together, our findings uncover a conserved Yki/YAP-Src42A/SRC positive feedback loop promoting tumor cell migration and provide SRC as a potential therapeutic target for YAP-driven metastatic tumors.

## Introduction

The Hippo pathway controls organ size by coordinating cell proliferation and cell apoptosis through the transcriptional coactivator Yki in *Drosophila* and YAP/TAZ in mammals [1–5]. Deregulation of the Hippo pathway has been linked to numerous human disorders, including cancers [4–6]. The Hippo pathway consists of a core kinase cascade, wherein the kinase Hpo phosphorylates and activates the downstream kinase Wts which, in turn, phosphorylates the transcriptional coactivator Yki on multiple serine residues to prevent its nuclear accumulation [7, 8]. In the nucleus, Yki pairs with the DNA-binding transcriptional factor Sd to drive target gene expression [9–11]. Well-documented Yki/YAP/TAZ target genes include *ex* [12], *cycE* [13], *myc* [14, 15], *ban* [16], *e2f1* [17], *CTGF* [11], and *diap1* [13], which play pro-proliferative or anti-apoptotic roles. Target genes of Yki/YAP in other cellular processes, such as cell migration, are much less understood. Identifying novel targets downstream of Yki/YAP can not only deepen our understanding of the Hippo pathway, but also pave ways for novel treatment of Hippo-related cancers.

Activation of YAP/TAZ, as manifested by genomic locus amplification, gene fusion, increased expression, or enhanced nuclear translocation, is frequently observed in malignant tumors [5, 6, 18]. These observations strongly suggest that YAP/TAZ activation contributes to tumor progression and metastasis. Indeed, overexpression of YAP in nontransformed epithelial cells results in epithelial-to-mesenchymal transition (EMT), a critical process for cancer metastasis [19, 20]. In addition, ectopic expression of YAP can promote cell migration and invasion in cultured tumor cell line [21, 22]. The role of YAP/TAZ in promoting metastasis has been further implicated in several cancers [18]. Consistently, in *Drosophila* ovary, overexpression of *hpo* disrupts polarization of the actin cytoskeleton and thus attenuates border cell migration [23]. How Yki/YAP controls the key steps of metastasis, such as cell migration, remains poorly understood.

To explore the role of Hippo signaling in tumor cell migration, we employed an excellent *Drosophila* tumor model, in which RNAi of the neoplastic tumor suppressor gene *scrib* confers wing imaginal disc cells invasive characteristics [24]. We found that loss of *hpo* or *wts* enhanced *scrib*-RNAi-induced cell migration and this enhancement was mediated by Yki. Furthermore, we identified *src42A*, a *Drosophila* homolog of human oncogene *SRC*, as a direct target of Yki which is dispensable for Yki to promote tumor cell migration. Yki, through its DNA-binding partner Sd, directly binds to the second intron of *src42A* to drive its transcription. Intriguingly, Src42A enhances Yki transcriptional activity vice versa, forming a positive feedback loop. Finally, we showed that SRC is also a target of YAP in human hepatocellular carcinoma (HCC) cells and responsible for YAP-induced cell metastasis. Taken together, we unveiled Src42A/SRC is a critical transcriptional target for Yki/YAP-induced tumor cell migration, and provided SRC as a potential therapeutic target for Hippo-related cancers.

## Results

### Inhibition of the Hippo pathway promotes tumor cell migration

**Fig. 1:**
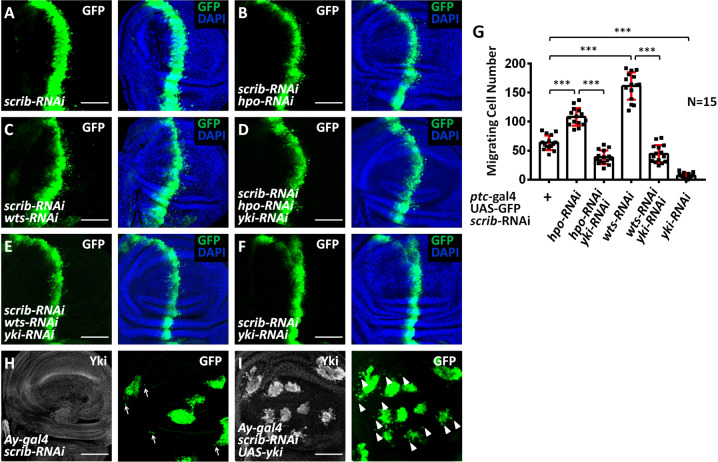
Inhibition of the Hippo pathway promotes tumor cell migration through Yki. **A** Knockdown of *scrib* in the wing disc triggered cell migration from A/P boundary to posterior. GFP (green) marks the expression pattern of *ptc-gal4* driver. **B** Knockdown of *hpo* increased *scrib*-RNAi-induced cell migration. **C** Silence of *wts* elevated *scrib*-RNAi-induced cell migration. **D** A wing disc expressing *hpo* RNAi plus *yki* RNAi simultaneously under *scrib* RNAi background were stained with GFP (green) and DAPI (blue). Knockdown of *yki* attenuated *hpo*-RNAi-enhanced cell migration. **E** Knockdown of *yki* attenuated *wts*-RNAi-enhanced tumor cell migration. **F** Knockdown of *yki* effectively blocked *scrib*-RNAi-induced cell migration. **G** Quantification of migrating cell numbers in *A*-*F* (*N* = 15). Data are presented as means ± SD of values from fifteen wing disks. **H** A wing disc expressing *scrib* RNAi by *Ay-gal4* was stained to show Yki (white) and GFP (green). Knockdown of *scrib* triggered moderate cell migration (arrows). **I** A wing disc simultaneously expressing *scrib* RNAi and *yki* induced apparent cell migration (arrowheads). Scale bars: 50 μm for all images.

In *Drosophila* wing disks, knockdown of *scrib* along the anterior/posterior (A/P) boundary using the *ptc-gal4* driver induces an invasive cell migration phenotype, which has been widely used as model tumor cell migration [25]. To investigate whether the Hippo pathway is involved in regulating tumor cell migration, we genetically manipulated this pathway activity in this tumor cell migration model. Compared with the control disc (Fig. 1A, G), knockdown of *hpo* or *wts* enhanced the migration of *scrib*-RNAi cells (Fig. 1B, C, G). However, lacZ co-expression did not affect cell migration (Fig. S1A–C), removing the effect of Gal4 titration. This enhancement was mediated by Yki, as silencing *yki* abolished *hpo* or *wts*-RNAi-induced enhancement of tumor cell migration (Fig. 1D, E, G). In contrast, knockdown of *yki* nearly blocked *scrib*-RNAi-induced cell migration (Fig. 1F, G). To confirm the RNAi efficiencies, we dissected the salivary gland for RT-qPCR assays. The immunostaining results showed that the *ptc*-gal4 was able to drive gene expression throughout the salivary gland (Fig. S1D, E). RT-qPCR data revealed that RNAi lines could silence corresponding genes (Fig. S1F). To show that the role of Yki in *scrib*-RNAi-induced cell migration is not confined to the *ptc*-expressing cells, we created random *scrib* RNAi clones using the FLP-out technique which were marked with green fluorescence (GFP) [26]. Knockdown of *scrib* induced moderate cell migration in these clones (Fig. 1H), which was enhanced by overexpressing *yki* (Fig. 1I). Taken together, these results indicate that inhibition of the Hippo pathway promotes *scrib*-RNAi-induced tumor cell migration.

### *src42A* is a transcriptional target of Yki

To identify the related target gene(s) accounting for Yki-enhanced tumor cell migration, an RNA-seq analysis was carried out. We collected adult heads of control (*GMR*) and *yki* overexpression (*GMR* > *yki*) flies for RNA-seq analysis. Compared with the control, 1241 genes were upregulated in *GMR* > *yki* samples (Fig. S2A). By comparing our RNA-seq results with other published RNA-seq [27] and Yki ChIP-seq data [28], we found 123 overlapping genes (Fig. S2B). The list of overlapping genes encompasses several well-known Yki-Sd targets, such as *ex* [12], *wg* [29], *rho1* [30], *ds* [31], and *crb* [32] (Fig. S2C), confirming the reliability of this analysis. We selected *src42A* (Fig. S2C), which is a *Drosophila* homolog of human oncogene *SRC*, for further investigation given its capability of promoting tumor metastasis [33, 34].

To validate our RNA-seq results, we used RT-qPCR analysis to examine the expression level of *src42A* as well as several other target genes. Consistent with the RNA-seq result, elevated *src42A* expression was detected by RT-qPCR in *GMR* > *yki* samples (Fig. S2D). In addition, analysis of published ChIP-seq data revealed robust enrichment of Yki on the genomic locus of *src42A* (Fig. S2F) as well as the known Yki target *ex* (Fig. S2E) [28]. Taken together, these results suggest that *src42A* is a potential transcriptional target of Yki.

**Fig. 2:**
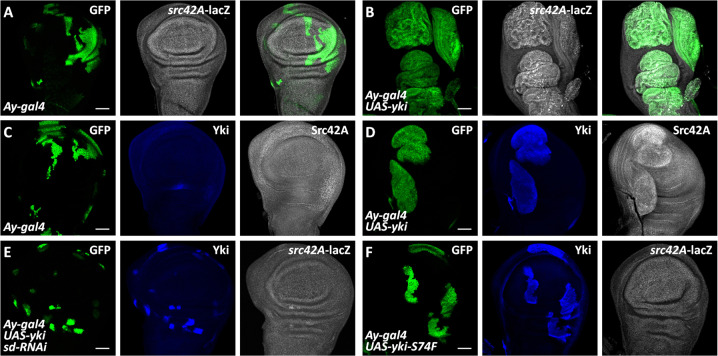
Yki activates *src42A* transcription. **A** A control wing disc expressing GFP via *Ay-gal4* was stained to show GFP (green), Yki (blue) and *src42A*-lacZ (white). Notably, *src42A*-lacZ evenly expresses in the wing disc. **B** Overexpression of *yki* apparently promoted *src42A*-lacZ expression. **C** A control wing disc was stained with GFP (green), Yki (blue), and Src42A (white). **D** Ectopic expression of *yki* by *Ay-gal4* increased Src42A protein level. **E** A wing disc simultaneously expressing *yki* and *sd* RNAi was stained to show GFP (green), Yki (blue) and *src42A*-lacZ (white). Knockdown of *sd* effectively suppressed Yki-induced *src42A*-lacZ upregulation. **F** Yki-S74F failed to activate *src42A*-lacZ expression in the wing disc. Scale bars: 50 μm for all images.

To confirm that *src42A* is a bona fide target of Yki, we used a *src42A*-lacZ reporter, in which the lacZ sequence was inserted downstream of *src42A* promoter. Compared with the control disc (Fig. 2A), overexpression of *yki* increased *src42A*-lacZ level (Fig. 2B). Secondly, we generated mouse anti-Src42A antibody to verify the induction of endogenous Src421A by Yki. Overexpression of a dominant negative form of *src42A* (*src42A*^*DN*^) elevated (Fig. S3A, B), while knockdown of *src42A* decreased staining signals (Fig. S3C), confirming the specificity of the antibody. Consistently, ectopic expression of *yki* also upregulated Src42A protein level (Fig. 2D), compared to the control disc (Fig. 2C).

### Yki activates src42A expression through Yki-Sd complex

As a transcriptional coactivator, Yki acts with the DNA-binding partner Sd to drive gene expression [9, 10]. We next examined whether Sd is required for Yki to induce *src42A* expression. Compared with *yki* overexpression alone (Fig. 2B), simultaneous knockdown of *sd* repressed *src42A*-lacZ upregulation (Fig. 2E). A previous study showed that mutation of S94 of YAP abolishes its affinity to TEAD1 [35]. Intriguingly, this serine residue was evolutionarily conserved between *Drosophila* Yki and human YAP (Fig. S4A). Consistently, Yki-S74F with the conserved serine substituted by phenylalanine (F) indeed lost its binding activity to Sd (Fig. S4B, C). We next further assessed the Yki-S74F mutant by examining two well accepted transcriptional reporters of Yki-Sd complex. To ensure equal expression of Yki and Yki-S74F transgenes, UAS-*yki* and UAS-*yki*-S74F constructs were introduced into the same genomic locus using the phiC31 integrase system [36]. Compared with control disks (Fig. S4D, G), overexpression of *yki* markedly increased the expression levels of two reporters *diap1*-lacZ (Fig. S4E) and *ex*-lacZ (Fig. S4H), while Yki-S74F failed to do so (Fig. S4F, I), indicating that Yki-S74F is not able to interact with Sd to drive target gene expression. In keeping with the *sd* RNAi results (Fig. 2E), the Yki-S74F mutant was also not able to induce the expression of *src42A*-lacZ (Fig. 2F). Taken together, these results have clearly demonstrated that Yki activates *src42A* expression in an Sd-dependent manner.

### Src42A is required for Yki to induce tumor cell migration

**Fig. 3:**
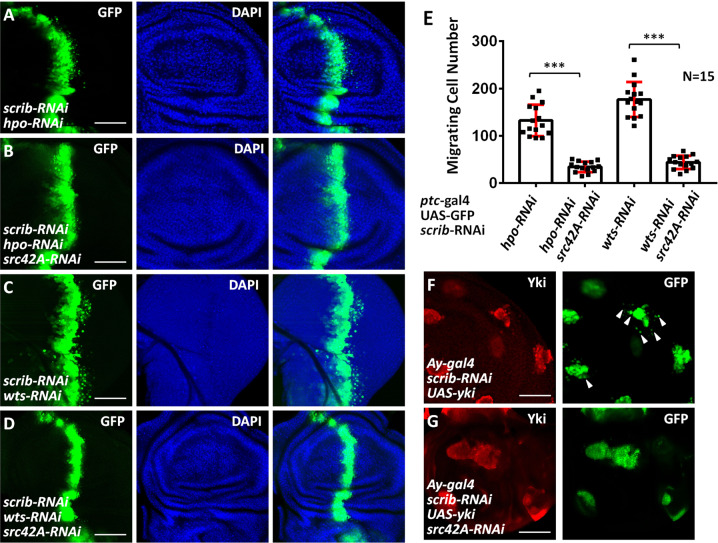
Src42A is required for Yki to promote tumor cell migration. **A** Knockdown of *hpo* under *scrib* RNAi background caused cell migration. **B** Knockdown of *src42A* inhibited *hpo*-RNAi-induced cell migration under *scrib* RNAi background. **C** Silence of *wts* under *scrib* RNAi background triggered robust cell migration. **D** Silence of *src42A* decreased *wts*-RNAi-induced cell migration under *scrib* RNAi background. **E** Quantification of migrating cell numbers in **A**–**D** (*N* = 15). Data are presented as means ± SD of values from fifteen wing disks. **F** A wing disc expressing *scrib* RNAi and UAS-*yki* by *Ay-gal4* was stained to show Yki (red) and GFP (green). Migrating cells are marker by arrowheads. **G** Knockdown of *src42A* suppressed Yki-induced tumor cell migration. Scale bars: 50 μm for all images.

Having established *src42A* as a transcriptional target of Yki, we then assessed the functional relevance of Src42A in Yki-mediated tumor cell migration. Compared with *hpo* RNAi alone (Fig. 3A, E), simultaneous knockdown of *src42A* inhibited tumor cell migration (Fig. 3B, E). Similarly, silence of *src42A* also reduced *wts*-RNAi-induced tumor cell migration (Fig. 3C–E). Furthermore, enhanced *scrib*-RNAi-induced cell migration by *yki* overexpressing (Fig. 3F) was suppressed by *src42A* knockdown (Fig. 3G). Taken together, these results suggest Src42A is a critical downstream effector of Yki in tumor cell migration.

### Sd binds directly to the second intron of *src42A*

**Fig. 4:**
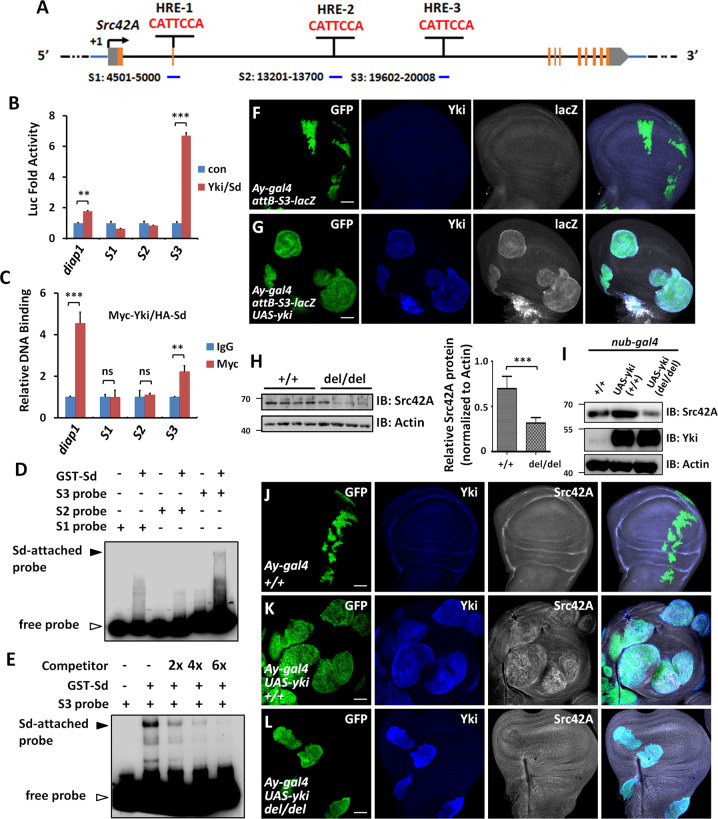
*src42A* is a direct transcriptional target of Yki-Sd. **A** Schematic view of *src42A* locus showed three presumptive HRE sites (CATTCCA), one in the second exon and the others in the second intron. Luciferase reporters and lacZ reporters used in subsequent experiments were connected to S1, S2, and S3 fragments. The numbers behind S1, S2, and S3 represent the start sites and end sites of indicated fragments (the transcription start site of *src42A* is designed as +1). **B** Transfection of Yki and Sd increased S3-Luc activity in S2 cells. The *diap1*-Luc acts as a positive control. **C** ChIP-qPCR analysis showed Myc-Yki enrichment on the third HRE region of *src42A* gene in S2 cells. The *diap1* promoter region serves as a positive control. **D** EMSA analysis revealed that GST-Sd protein exclusively bound to the S3 probe. **E** Biotin free S3 fragments were able to compete with biotin-labeled S3 probe to interact with GST-Sd protein. **F** A control wing disc was stained to show the expression of GFP (green), Yki (blue) and S3-lacZ (white). S3-lacZ showed basal expression throughout the wing disc. **G** Ectopic expression of *yki* apparently elevated S3-lacZ signals. **H** IB experiments showed that the third HRE deletion decreased Src42A protein. Quantification was shown on the right. **I** IB assays showed that the third HRE deletion mutant could not respond to Yki. For each panel, fifty wing disks were dissected for protein extraction. Actin serves as a loading control. **J** A control wing disc expressing GFP by *Ay-gal4* was stained to show GFP (green), Yki (blue) and Src42A (white). **K** A wing disc expressing *yki* under wild type background was stained with GFP (green), Yki (blue) and Src42A (white). Yki apparently promoted *src42A* expression. **L** A wing disc expressing *yki* under *src42A* mutant background was stained with GFP (green), Yki (blue), and Src42A (white). Yki failed to activate *src42A* when the third HRE was deleted. Scale bars: 50 μm for all images.

Since Sd is indispensable for Yki-induced *src42A* expression, we next examined whether *src42A* is a direct transcriptional target of Yki-Sd complex. Sd recognizes a conserved DNA sequence, named as Hippo responsive element (HRE), to turn on gene expression [9]. We searched in the *src42A* gene and found three potential HREs (named as HRE-1, HRE-2, and HRE-3), of which HRE-1 is localized in exon region, while HRE-2 and HRE-3 are localized in the second intron region (Fig. 4A). To test which HRE is involved in regulating *src42A* expression, we performed luciferase analyses, in which Yki-Sd complex was used to drive luciferase expression from a DNA fragment containing one of these three HREs (Fig. 4A). Interestingly, only S3-Luc which contains HRE-3 responded to Yki-Sd (Fig. 4B). To check whether Yki-Sd directly binds to HRE-3, we carried out ChIP-qPCR and found that Yki indeed bound to HRE-3 in the presence of Sd (Fig. 4C). In addition, electrophoretic mobility-shift assays (EMSAs) confirmed the direct interaction between Sd and HRE-3 (Fig. 4D, E). To further validate this Sd binding site in vivo, we generated lacZ reporters, in which the lacZ sequence was inserted downstream of the DNA fragment containing one of these three HREs (Fig. 4A). S1-lacZ, S2-lacZ, and S3-lacZ were introduced into the same locus using the phiC31 integrase system. Compared with control disks (Fig. S5A, C), overexpression of *yki* was unable to activate S1-lacZ and S2-lacZ expression (Fig. S5B, D). However, Yki sharply turned on S3-lacZ expression (Fig. 4G), compared to the control disc (Fig. 4F).

After demonstrating that Sd directly binds to the third HRE, we sought to examine whether this HRE is essential for Yki to induce endogenous *src42A* expression. To this end, the Cas9-mediated genome editing was employed to delete the third HRE. Two guide RNAs (gRNAs) and Cas9 plasmid were injected into embryos (Fig. S5E). After screening, we identified a 241 bp deletion line of *Drosophila*, in which the third HRE together with part of the adjacent sequence were deleted without affecting the coding sequence (Fig. S5F, G). The homozygous mutants (refers to del/del hereafter) could survive to adulthood and did not show any obvious developmental defects. Intriguingly, compared with wild type controls, del/del flies showed a decreased expression of *src42A* (Fig. 4H). In addition, overexpression of *yki* elevated Src42A protein in wild type background, but not in del/del *src42A* mutants (Fig. 4I–L). Together, our results reveal that Yki-Sd complex directly binds to HRE-3 to drive the expression of *src42A*.

### Src42A inhibits the Hippo pathway activity

**Fig. 5:**
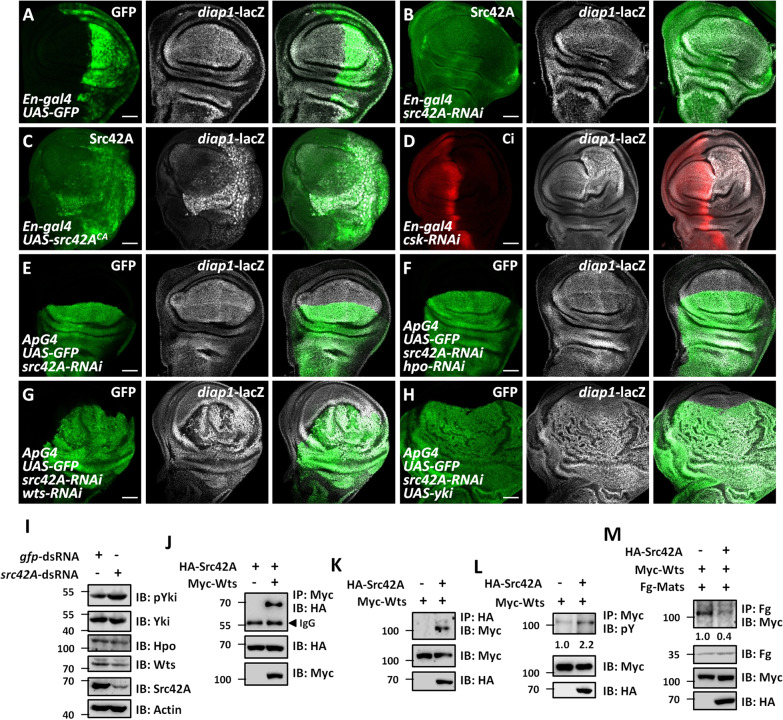
Src42A suppresses the Hippo pathway activity. **A** A control wing disc was stained to show GFP (green) and *diap1*-lacZ (white). *En-gal4* drives gene expression in the posterior region of the wing disc. **B** A wing disc expressing *src42A* RNAi by *En-gal4* was stained to show Src42A (green) and *diap1*-lacZ (white). Of note, knockdown of *src42A* decreased *diap1*-lacZ. **C** Overexpression of a constitutive active form of Src42A (Src42A^CA^) elevated *diap1*-lacZ in the wing disc. **D** Knockdown of *csk* in the wing disc activated *diap1*-lacZ expression. **E** A control wing disc expressing *src42A* RNAi by *ApG4* was stained to show GFP (green) and *diap1*-lacZ (white). *ApG4* exclusively expresses in the dorsal region of wing disc. **F** Knockdown of *hpo* failed to restore the decreased *diap1*-lacZ caused by *src42A* RNAi. **G** Silence of *wts* upregulated *diap1*-lacZ under *src42A* RNAi background. **H** Ectopic expression of *yki* rescued *src42A*-RNAi-induced *diap1*-lacZ decrease. **I** Knockdown of *src42A* increased pYki level in S2 cells. Actin serves as a loading control. **J** Myc-Wts pulled down HA-Src42A in S2 cells. **K** HA-Src42A pulled down Myc-Wts in S2 cells. **L** Src42A promoted tyrosine phosphorylation of Wts in S2 cells. **M** Src42A decreased the affinity between Wts and Mats in S2 cells. Scale bars: 50 μm for all images. All western blot assays were repeated three times. The numbers on **L** and 0y9t 660 7p ik>.'p represented the relative intensities of bands.

The *src42A* gene encodes a non-receptor tyrosine kinase involved in multiple cellular processes, such as cell proliferation [37], cell adhesion [38], cell death [34], and regeneration [39]. Its mammalian orthologue *SRC* is the first identified oncogene [40]. Recently, several studies have shown that SRC is involved in mammalian Hippo pathway regulation. SRC inhibits the Hippo pathway through tyrosine phosphorylation of LATS1 (the *Drosophila* Wts homolog) to prevent LATS1 activation [41]. Besides, SRC activates the Rho/ROCK pathway to influence actin cytoskeleton, in turn suppress LATS activity [42]. In addition, SRC can also directly phosphorylate YAP to enhance its stability and transcriptional activity [43, 44]. Thus, we next explored whether Src42A regulates Yki activity to form a feedback loop in *Drosophila*. Compared with the control disc (Fig. 5A), knockdown of *src42A* slightly but detectably, decreased *diap1*-lacZ signals (Fig. 5B). Conversely, ectopic expression of a constitutive active form of Src42A (Src42A^CA^) promoted *diap1*-lacZ expression (Fig. 5C). In addition, depletion of Src42A inhibitor, the C-terminal Src kinase (Csk) [45] also elevated *diap1*-lacZ level (Fig. 5D). These data show that Src42A is a positive regulator of Yki activity.

To elucidate the mechanism whereby Src42A regulates the Hippo pathway, we performed epistatic analyses. Compared with *src42A* RNAi alone (Fig. 5E), simultaneous knockdown of *hpo* failed to restore *diap1*-lacZ expression (Fig. 5F). However, knockdown of *wts* (Fig. 5G) or overexpression of *yki* (Fig. 5H) rescued *src42A*-RNAi-induced *diap1*-lacZ downregulation, suggesting that Src42A acts upstream of or parallel to Wts.

The Hippo pathway is a kinase cascade that ultimately leads to Yki phosphorylation and cytoplasmic retention [5]. Thus, the phosphorylation status of Yki protein can reflect the Hippo pathway activity [46]. Knockdown of *src42A* in S2 cells elevated phosphorylated Yki (pYki) level (Fig. 5I). However, we also observed that knockdown of *src42A* decreased Wts protein, without affecting Yki and Hpo protein levels (Fig. S6A). Considering the previous study has demonstrated that *wts* is a potential transcription target of Yki [28], it is acceptable that knockdown of *src42A* reduces Wts through inhibiting *wts* transcription.

Since the non-receptor tyrosine kinase Src42A/SRC plays its roles always through binding and phosphorylating substrates [47, 48], we expected that Src42A possibly interacted with a component of the Hippo pathway. To test this possibility, co-immunoprecipitation (co-IP) assays were carried out in *Drosophila* S2 cells. Consistent with above epistatic results, Src42A exclusively interacted with Wts (Fig. 5J, K), but not with other Hippo pathway components (Fig. S6B–J). Furthermore, Src42A promoted Wts tyrosine phosphorylation (Fig. 5L). During Hippo signal transduction, the physical interaction between Wts and its adapter, Mob as a tumor suppressor (Mats), is a critical step for Wts kinase activation [49]. In agreement with the previous observation in mammalian cells [41], we found that the affinity between Wts and Mats was weakened by Src42A (Fig. 5M), indicating that the mechanism whereby Src42A/SRC regulates Hippo signaling through Wts/LATS1 is conserved from *Drosophila* to mammalian cells.

### SRC is a target of YAP and is critical for YAP-induced tumor cell migration

**Fig. 6:**
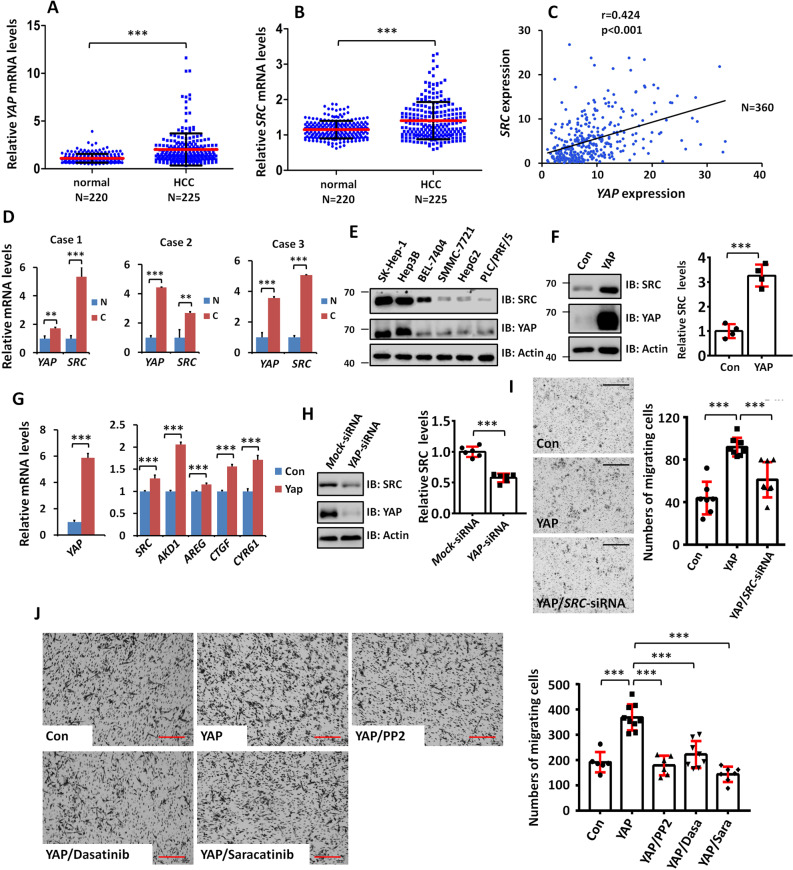
YAP promotes HCC cell migration through SRC. **A** Comparison of normal liver tissues (*N* = 220) with HCC samples (*N* = 225) showed upregulation of *YAP* mRNA in HCC samples. **B** The expression of *SRC* was increased in HCC samples. **C** Correlation analysis of *SRC* and *YAP* expression in HCC samples (*N* = 360) revealed positive correlation between *SRC* and *YAP*. **D** RT-qPCR analyses showed upregulation of *YAP* and *SRC* expression in clinical HCC samples. **E** Immunoblots (IBs) analyses for protein levels of YAP and SRC in six HCC cell lines. PLC/PRF/5 cells showed minimum expression of SRC and YAP. Actin acts as a loading control. **F** Transfection of YAP increased SRC protein in PLC/PRF/5 cells. Actin serves as a loading control. Quantification was shown on the right. **G** RT-qPCR analyses showed YAP-dependent upregulation *SRC* mRNA level in PLC/PRF/5 cells. *AKD1*, *AREG*, *CTGF*, and *CYR61* are well-known YAP targets and used as positive controls. **H** Knockdown of *YAP* downregulated SRC protein level in PLC/PRF/5 cells. Actin serves as a loading control. Quantification was shown on the right. **I** Transwell analyses showed that YAP promoted PLC/PRF/5 cell migration, which was attenuated by *SRC* knockdown. Quantification was shown on the right. **J** The increased cell migration induced by YAP was suppressed by several SRC inhibitors in SK-Hep-1 cells. Quantification was shown on the right. Scale bars: 600 μm for all images.

Having shown that the Yki-Src42A module promotes tumor cell migration in *Drosophila*, we then tested whether a similar mechanism operates in human tumor cells. We chose human hepatocellular carcinoma (HCC) because our previous study has revealed that YAP protein is upregulated in HCC samples and closely linked to tumor progression [50]. To assess the expression of YAP and SRC in HCC, we first analyzed the microarray data from the Oncomine database (https://www.oncomine.org) and found that both *YAP* and *SRC* were highly expressed in HCC tissues compared with normal liver tissues (Fig. 6A, B) [51]. A co-expression analysis using protein atlas data (https://www.proteinatlas.org) showed that *SRC* mRNA levels positively correlated with *YAP* mRNA in HCC samples (Fig. 6C). To validate these bioinformatic results, we carried out RT-qPCR experiments and found that both *YAP* and *SRC* were indeed upregulated in HCC samples compared to the adjacent normal tissues (Fig. 6D).

To test whether *SRC* is a target of YAP in HCC cells, we first analyzed protein levels of SRC and YAP in six HCC cell lines. Both RT-qPCR and western blot results revealed that SRC positively correlated with YAP in different HCC cell lines (Fig. 6E and Fig. S7A). We then tried to manipulate YAP level in cultured HCC cells. In order to reduce the interference of endogenous YAP, PLC/PRF/5 cells with low YAP expression were selected for subsequent studies. In line with our idea, transfection of YAP substantially elevated both SRC protein (Fig. 6F) and *SRC* mRNA (Fig. 6G) in PLC/PRF/5 cells. Conversely, knockdown of YAP inhibited SRC expression (Fig. 6H). In mammalian cells, YAP activates target gene expression through its partner TEAD [5]. To test whether YAP turns on *SRC* expression through YAP-TEAD complex, we sought to silence TEAD. The mammalian genome contains four highly homologous TEAD family members: TEAD1–4. To overcome the functional redundancy, we attempted to block the activity of all TEAD factors by treating cells with verteporfin (VP), a compound is capable of blocking YAP-TEADs interactions [52]. YAP-5SA, the active form of YAP [53], was able to increase SRC protein level in HepG2 cells, which was attenuated by VP treatment (Fig. S7B). Furthermore, we generated a mutant form of YAP, YAP-5SA-S94F, which failed to bind TEAD factors (Fig. S7C). Compared with YAP-5SA, YAP-5SA-S94F was unable to increase SRC protein level (Fig. S7D). The above results together suggest that YAP activates *SRC* expression through YAP-TEAD complex.

Moreover, we searched TEAD binding sites in *SRC* gene region and found three sites (HRE-1, HRE-2, and HRE-3) (Fig. S7E). Intriguingly, all three HREs localized in the first intron (Fig. S7E). We generated luciferase reporters, which respectively contain one HRE. The results showed that HRE-1-Luc and HRE-3-Luc were able to respond to YAP-5SA in both 293T cells (Fig. S7F) and SMMC-7221 cells (Fig. S7G). Consistently, ChIP-qPCR assay revealed that Myc-YAP-5SA could pull down HRE-1 and HRE-3, not HRE-2 in SMMC-7721 cells (Fig. S7H). Taken together, these results suggest that YAP/TEAD activates *SRC* expression through direct binding the HRE-1 and HRE-3 degrons.

Given our *Drosophila* data demonstrating that Yki promotes tumor cell migration through its target Src42A, we next examined whether SRC is also involved in YAP-induced HCC cell migration. Using transwell assays, we found that YAP promoted PLC/PRF/5 cell migration, which was attenuated by *SRC* knockdown (Fig. 6I), indicating that YAP enhances tumor cell migration, at least partially through SRC. In fact, several inhibitors of SRC have been under preclinical anticancer experiments [54, 55]. We next examined whether these inhibitors are able to suppress YAP-induced HCC cell migration. Compared with control cells, transfection of YAP apparently promoted cell migration, which was blocked by treatment with SRC inhibitors, including PP2, Dasatinib (Dasa) and Saracatinib (Sara) (Fig. 6J). Taken together, our results show that *SRC* is a transcriptional target of YAP in HCC cells and SRC inhibitors are able to suppress YAP-induced tumor cell migration.

## Discussion

Over the past decade, the Hippo pathway effector YAP has been shown to be frequently activated in malignant tumors and has emerged as an important player in cancer initiation, progression and metastasis. The well-known function of YAP in promoting cell proliferation and inhibiting apoptosis has been linked to its potent role in driving the initiation and growth of tumors. In contrast, how YAP activation contributes to tumor metastasis is still poorly understood. In this study, we found that Yki directly induces the transcription of *src42A*, a homolog of human oncogene *SRC*, to promote cell migration, a key step of tumor metastasis. We went further to show that *SRC* is also a target of YAP and *SRC* induction by YAP contributes to the migration of HCC cells. Interestingly, Src42A/SRC is able to activate Yki/YAP, thus forming a feedforward loop to drive tumor progression. Taken together, our study identifies a conserved Yki/YAP-Src42A/SRC positive feedback loop promoting tumor cell migration and provides SRC as a potential therapeutic target for YAP-related malignancies.

Although it is clear that the Hippo pathway regulates growth and apoptosis, its role in cell migration is still unclear. Two previous studies showed that the Hippo pathway promotes the migration and extrusion of noncancerous cells through distinct mechanisms [56, 57]. Herein, we showed that the Hippo pathway restricts the activity of Yki to suppress the migration of tumor cells. Our results are consistent with the abundant evidence showing that Yki homolog YAP/TAZ promotes the migration and invasion of cancer cells [6, 18, 58]. Thus, whether Hippo signaling promotes or prevents cell migration might depend on cell transformation status. Interestingly, a recent study shows that YAP knockdown increases the migration of breast cancer cells [59]. Our results show that YAP promotes the migration of HCC cells. Thus, the role of YAP in cancer cell migration seems to be also context-dependent and requires more investigation.

So far, most of reported Yki transcriptional targets are pro-proliferative or anti-apoptotic genes [28]. The key to elucidate the role of the Hippo pathway in cell migration is to identify migration-related targets of Yki. Here, we demonstrate that *src42A* is a novel transcriptional target of Yki, and provide strong evidence to support Src42A is a critical effector for Yki-induced tumor cell migration. Our conclusion is firmly supported by the identification of a HRE in the second intron of *src42A* gene and by showing that this HRE is not only essential but also sufficient for Yki to induce *src42A* expression. The *SRC* oncogene has been strongly implicated in the initiation and progression of various human cancers. *SRC* gene encodes a non-receptor protein tyrosine kinase that phosphorylates several downstream targets, including STAT3, FAK, and RAS to regulate many cellular processes such as cell growth, invasion, angiogenesis and migration [60]. Elevated SRC protein or kinase activity has been reported in many human cancers. Although it is clear that the activity of SRC is controlled by phosphorylation, myristoylation and partner interaction, how to upregulate its expression in tumor cells still remain unknown. Here, we reveal that *SRC* is a transcriptional target of YAP in HCC cells.

Strict regulation of the Hippo pathway activity is essential for animal development and adult tissue homeostasis [1, 2, 4, 5, 61]. To achieve this goal, multiple negative feedback regulatory mechanisms have been employed to ensure the homeostasis of the Hippo pathway. For example, in *Drosophila*, several components of the Hippo pathway, including Ex and Ds, are Yki transcriptional targets [12, 31]. In turn, Ex and Ds activate Hippo signaling to inhibit Yki activity to form negative feedback loops [62, 63]. In human ovarian surface epithelial cells, YAP activates its upstream inhibitory kinase LATS2 through inducing *LATS2* transcription [64]. However, in this study, we instead identify a positive feedback loop to modulate the Hippo pathway: Yki activates *src42A* expression, and in turn, Src42A increases Yki transcriptional activity. We show that *SRC* is also a target of YAP in HCC. Several studies have demonstrated that SRC activates YAP in mammalian cells [41, 65], implying that this positive feedback mechanism is conserved in mammals. Although this study showed that Src42A activates Yki through phosphorylating Wts, we could not remove other mechanisms for Src42A regulating Yki. Our results suggest that through this positive feedback loop, Yki/YAP activity is amplified to drive tumor cell migration. It will be interesting to examine whether this positive feedback loop drives tissue growth in regeneration and tumorigenesis.

Although numerous studies have established an important role for the Hippo pathway in tumorigenesis, drugs targeting this pathway have been shown to be difficult due to a number of reasons. First, most of the key components of the Hippo pathway are tumor suppressors, which are not suitable targets for tumor therapy. Second, the downstream effector YAP is a transcriptional coactivator which is hard to be targeted by drugs. Third, YAP also plays an indispensable role in adult homeostasis and most of its known targets are necessary to maintain physiological cell growth and apoptosis. Therefore, the search for tumor-related genes downstream of YAP, is urgent for drug development. Here, we show that the oncogene *src42A*/*SRC* is a downstream target of Yki/YAP, and that SRC inhibitors are able to suppress YAP-induced tumor cell migration, providing SRC as an alternative target for Hippo-related cancer treatment. In addition to HCC, dysregulation of the Hippo pathway has been linked to various other types of cancers, such as colon cancer [66] and breast cancer [67]. It is thus worthwhile to examine whether targeting this YAP-SRC positive feedback loop also suppresses tumor cell migration in other types of malignancies.

## Materials and methods

### Drosophila genetics

UAS-GFP, *hpo*-RNAi, *wts*-RNAi, *yki*-RNAi, UAS-*yki*, *Ay-gal4*, *ApG4*, *ptc-gal4*, *En-gal4*, and *diap1*-lacZ had been described in our previous study [50]. *src42A*-RNAi, *scrib*-RNAi and UAS-lacZ were kindly provided from Dr. Lei Xue lab [34]. *ex*-lacZ (#11067, BDSC), *csk*-RNAi (#41712, BDSC), *src42A*-lacZ (#102735, KDSC), UAS-*src42A*^*DN*^ (#109998, KDSC), UAS-*src42A*^*CA*^ (#108774, KDSC) and *sd*-RNAi (#101497, VDRC) were purchased from Bloomington *Drosophila* Stock Center (BDSC), Kyoto *Drosophila* Stock Center (KDSC) or Vienna *Drosophila* Stock Center (VDRC). Transgenic flies expressing equal Yki and Yki-S74F were generated by inserting UAS-attB-*yki* and UAS-attB-*yki*-S74F constructs into the 68A4 attP locus (#25710, BDSC) using phiC31 integration system. To make lacZ reporter flies, we first amplified S1 (4501–5000), S2 (13201–13700) and S3 (19602–20008) sequences and respectively cloned into CPLZN, an enhancer vector that contains the *hsp70* basal promoter and lacZ reporter [9]. Then, these recombinant plasmids were introduced into 25C6 attP locus (#25709, BDSC) respectively, to get attB-S1-lacZ, attB-S2-lacZ and attB-S3-lacZ flies. To generate the third HRE site deleted *src42A* fly (*scr42A*^del/del^), Cas9-mediated genome editing technique was performed according to the previous described [68]. In brief, two gRNAs were designed using an on-line website (http://crispr.dbcls.jp/), and were inserted downstream of *U6b* promoter [68] to get *U6b*-gRNA-1 and *U6b*-gRNA-2 constructs. Then, these two *U6b*-gRNA constructs (250 ng/μL for each) were co-injected with *nos*-Cas9 plasmid (500 ng/μL) into *w*^*1118*^ embryos. All G0 adult flies from injected embryos were crossed to balancer (Sp/CyO) flies to get F1 progenies. Each F1 male flies were crossed to female balancers to establish strains. Genomic DNA was extracted from each strain for RCR amplification. PCR products were sent for Sanger sequencing to identify the third HRE site deleted strain. Through screening, we identified a 241 bp deletion *Drosophila* line, in which the third HRE and adjacent sequence were deleted but no effect on the coding sequence. The sequences of gRNAs were shown as following: gRNA-1: 5′-AAC GCG TTG GGT TAG AGC TA-3′ and gRNA-2: 5′-GGC TCG GGC AGC ACG TGT AT-3′. The primers for PCR amplification were as following: 5′-GTA AGT TTA ACC GTT CAG CT-3′ (forward) and 5′-CAA AGA AGT TCA TTA AGC GA-3′ (reverse). To knockdown or overexpress genes, the male flies of RNAi or transgenes were crossed with indicated *gal4* virgin flies at 25 °C. For FLP-out technique, larvae were under heat shock at 37 °C for 5 min at 36 h before dissection. All *Drosophila* stocks in this study were maintained and raised under standard conditions.

### Immunostaining

Immunostaining of imaginal disks were carried out with previous protocols [69, 70]. Third-instar larvae were dissected in phosphate buffered solution (PBS) and fixed in freshly made 4% formaldehyde in PBS at room temperature for 20 min, then washed three times with PBT (PBS supplied with 0.1% Triton X-100). Larvae were incubated overnight with indicated primary antibodies in PBT at 4 °C, then washed with PBT for three times and incubated with proper fluorophore-conjugated secondary antibodies for 2 h at room temperature. After washing three times in PBT, disks were separated and mounted in 40% glycerol. Images were captured with Zeiss confocal microscope. Antibodies used in this study were shown as follows: rat anti-Ci (1:50, DSHB); rabbit anti-β-Galactosidase (1:500, MBL), mouse anti-MMP1 (1:5, DSHB). Mouse anti-Yki (1:50) was generated using aa1-250 of Yki protein as the antigen [50]. Mouse anti-Src42A (1:50) was generated using aa1–350 from Src42A protein as the antigen. To mark cell nuclei, wing disks were stained with DAPI (1:1000, Sigma) for 15 min before mounting. Secondary antibodies used in this research were purchased from Jackson ImmunoResearch, and were diluted at 1:500.

### Cell culture and transfection

*Drosophila* S2 cells were maintained in serum-free insect cell culture medium (HyClone) supplemented with 1% penicillin/streptomycin (Sangon Biotech). Six human HCC cell lines (SK-Hep-1, Hep3B, BEL-7404, SMMC-7721, HepG2, and PLC/PRF/5) were purchased from the ATCC and cultured according to the standard protocols. All cell lines were examined for mycoplasma every one month using the Mycoplasma Detection kit (Vazyme). Cells were transfected using Exfect2000 (Vazyme) according to the manufacturer’s instructions. Most constructs used in transfection were mentioned in our previous study [50]. Myc-Yki-S74F was generated via PCR-based site-directed mutagenesis. dsRNA-mediated *src42A* knockdown in S2 cells was performed according to the previous described [50]. *src42A*-dsRNA targeted 1–700 bp of *src42A* gene. To silence indicated genes in HCC cells, siRNAs were transfected at a final concentration of 100 nM via lipo2000 (Invitrogen) according to the manufacturer’s instructions. The siRNA sequences were as follows: *YAP*-siRNA: 5′-GAC AUC UUC UGG UCA GAG AdTdT-3′; *SRC*-siRNA: 5′-GAC AGA CCU GUC CUU CAA GdTdT-3′. Forty-eight hours after transfection, cells were harvested for immunoprecipitation (IP) or immunoblotting (IB) analysis with standard protocols. For VP treatment, 5 μM VP incubates cells for 6 h before cell harvesting. The following antibodies were used in IP or IB: rabbit anti-pYki (1:5000), mouse anti-Yki (1:1000), rabbit anti-Hpo (1:5000), rabbit anti-Wts (1:5000), mouse anti-Src42A (1:1000), mouse anti-Actin (1:5000), mouse anti-Myc (1:2000 for IB, 1:200 for IP; Santa Cruz), mouse anti-HA (1:2000 for IB, 1:200 for IP; Santa Cruz), mouse anti-Fg (1:5000 for IB, 1:500 for IP; Sigma), rabbit anti-pan phospho-Tyrosine (pY) (1:1000; ABclonal), mouse anti-YAP (1:1000; Santa Cruz) and rabbit anti-SRC (1:1000; Affinity).

### RNA isolation and real-time quantitative PCR

Cells, HCC samples and adult *Drosophila* heads were lysed in TRIzol (Invitrogen) for RNA isolation following standard protocols. One microgram RNA was used for reverse transcription by HiScript^®^ Q RT SuperMix with gDNA wiper (Vazyme) according to the instructions. Real-time quantitative PCR (RT-qPCR) was performed on Bio-Rad CFX96^TM^ with ChamQ SYBR^®^ Color qPCR Master Mix (Vazyme). 2^−∆∆Ct^ method was used for relative quantification. The primer pairs used were as follows: *yki*, 5′-TGC CTA ATC GCT AAG ATA ATT C-3′ (forward) and 5′-CAG GTT GTT GGA CTT GAT C-3′ (reverse); *ex*, 5′-TCC TTG CTG AAA CAG ACT A-3′ (forward) and 5′-GGC TTA CGG TAG ATC CTT-3′ (reverse); *wg*, 5′-CTC CAT GTG GTG GGG CAT TG-3′ (forward) and 5′-CCA GTA CAC CGG GAT TGT CC-3′ (reverse); *rho1*, 5′-CTT GCC TTC TGA TTG TCT TC-3′ (forward) and 5′-GTC GTA GTC TGT CGT AGT C-3′ (reverse); *ds*, 5′-GAC ACA CCC TAA TTG TAA CC-3′ (forward) and 5′-CGC TGG CAT TAA CTT GAA-3′ (reverse); *crb*, 5′-GCA ACA ACA ACA GCA AGA-3′ (forward) and 5′-GAG GTA AGT GGA GCC ATT A-3′ (reverse); *src42A*, 5′-CTA TGC GTC AAC CTC TGC AA-3′ (forward) and 5′-TGG GGT CCA TTG TAC CAG AT-3′ (reverse); *actin*, 5′-GTA CCC CAT TGA GCA CGG TA-3′ (forward) and 5′-CGA ACA TGA TCT GGG TCA TC-3′ (reverse); *YAP*, 5′-GCA ACT CCA ACC AGC AGC AAC A-3′ (forward) and 5′-CGC AGC CTC TCC TTC TCC ATC TG-3′ (reverse); *SRC*, 5′-CAA GGT GAC CAT AGC CGA TGA-3′ (forward) and 5′-GGA CCG GAC ACT TTC CTG C-3′ (reverse); *AREG*, 5′-TCA CTT TCC GTC TTG TTT TGG-3′ (forward) and 5′-CGG GAG CCG ACT ATG ACT AC-3′ (reverse); *CYR61*, 5′-TAT TCA CAG GGT CTG CCC TC-3′ (forward) and 5′-AAC GAG GAC TGC AGC AAA A-3′ (reverse); *CTGF*, 5′-TAG GCT TGG AGA TTT TGG GA-3′ (forward) and 5′-GGT TAC CAA TGA CAA CGC CT-3′ (reverse); *AKD1*, 5′-GTG TAG CAC CAG ATC CAT CG-3′ (forward) and 5′-CGG TGA GAC TGA ACC GCT AT-3′ (reverse); *ACTIN*, 5′-GAT CAT TGC TCC TCC TGA GC-3′ (forward) and 5′-ACT CCT GCT TGC TGA TCC AC-3′ (reverse). Data are presented as means ± SD (standard deviation) of values from at least three experiments.

### Luciferase reporter assays

For luciferase experiments, the sequences containing potential HREs were amplified and inserted into pGL3-Basic-Luc vector [71] to generate S1-Luc (4501–5000), S2-Luc (13201-13700), S3-Luc (19602-20008), HRE-1-Luc (16145-16645), HRE-2-Luc (17285-17785), and HRE-3-Luc (17744-18244) reporters. Dual luciferase reporter assays were carried out according to the previous described [50]. *Drosophila* S2 cells or 293T cells transfected with indicated plasmids. Forty-eight hours after transfection, cells were lysed with passive lysis buffer and luciferase activities were measured using a Dual Luciferase Assay Kit (Vazyme) according to the manufacturer’s instructions. In this assay, *diap1*-Luc serves as a positive control. All luciferase activity data are presented as means ± SD of values from at least three experiments.

### ChIP-qPCR assays

For chromatin immunoprecipitation (ChIP) assay, S2 cells were transfected with indicated constructs. Forty-eight hours after transfection, cells were harvested and fixed with 4% fresh-made formaldehyde for 10 min at room temperature. After washed with PBS, cells were lysed with lysis buffer supplemented with protease inhibitor cocktail (#GK10014, Glpbio) and PMSF (#93482, Sigma). Then, the chromatin was sheared by sonication for proper cycles at 4 °C. The sheared chromatin was subjected for immunoprecipitation with mouse IgG (ABclonal) or anti-Myc antibody. After immunoprecipitation, the beads were washed three times with cell lysis buffer and treated with reverse cross-linking buffer supplemented with proteinase K (#RT403, Tiangen) at 65 °C for 6 h. The immunoprecipitated DNA was quantified using qPCR. Primers used in ChIP-qPCR were as follows: *diap1*, 5′-GCC CCG CCT TCA CTA AAA GTG-3′ (forward) and 5′-AAT TCT GTA AAC ATT TAA GG-3′ (reverse); S1, 5′-TTA AAT TAC GGT CTT TGT TTG-3′ (forward) and 5′-AAT TAA GAC TCG AGT TGT TC-3′ (reverse); S2, 5′-TTA AAC CGG TTC AAT TTG GG-3′ (forward) and 5′-GTG TGG GAG TTT TTT TTT TTT AC-3′ (reverse); S3, 5′-CTT TAA TTA ATG ATG ATG-3′ (forward) and 5′-AAG TGC CGT GGT TTC ACG AA-3′ (reverse); HRE-1, 5′-CCC AAG AAT CTT ATC CTC AG-3′ (forward) and 5′-GAG CTT TCG TGG GCC CTT CTC-3′ (reverse); HRE-2, 5′-CGT GAA CCC CGG GCG GGA GG-3′ (forward) and 5′-AAG GAA GGA GCC TGG GCT CC-3′ (reverse); HRE-3, 5′-CTA CAC CCA CCA CTG CTC TTC C-3′ (forward) and 5′-CGG CAG GGC ATC TAT CAG TGA TC-3′ (reverse). Data are presented as means ± SD of values from three experiments.

### Electrophoretic mobility shift assays

Biotin-labeled S1, S2, and S3 probes were synthesized using PCR-mediated DNA amplification. Biotin was tagged on the upstream of forward primers. The competitor DNA was synthesized using biotin free primers to amplify S3 sequence. Full-length Sd was subcloned into pGEX-4T-3 vector for expression as GST-Sd fusion protein in *E. coli*. The expressed GST-Sd protein was purified using GSH Sepharose column. Electrophoretic mobility shift assays (EMSA) were carried out using Chemiluminescent EMSA kit (Beyotime) according to the manufacturer’s instructions.

### Transwell assays

Transwell experiments were performed as described previously [66]. PLC/PRF/5 or SK-Hep-1 cells under indicated treatments were deprived of serum for 24 h before analyses. A total of 1 × 10^5^ cells were seeded to the upper chamber in transwell inserts (BD Biosciences) with serum-free medium, and medium containing 10% FBS was added into the lower chamber of 24-well plate (Corning). After additional 48 h, migrating cells adhered to the lower surface of filter were washed with PBS, fixed with 20% methanol for 20 min and stained with 0.1% crystal violet (Sangon Biotech). For SRC inhibitors treatment, cells were treated with PP2 (10 mM for 24 h, MedChemExpress) or Dasatinib (0.1 mM for 24 h, MedChemExpress) or Saracatinib (1 mM for 6 h, MedChemExpress) before seeding in chambers. The numbers of migrating cell per well were counted under a light microscope in eight predetermined fields. Data are presented as means ± SD of values from these eight fields.

### Patient samples

Fresh-frozen primary HCC tissues and their paired normal samples were obtained from patients undergoing surgical resection at Zhuhai People’s Hospital (Zhuhai, China) after consent was obtained from the patients. None of the patients received any prior radiochemotherapy. For total protein extraction, place the equal amount tissues in tubes and grind the tissue with a plastic rod for 50–60 times with twisting force on ice. Then, add five times cell lysis buffer (50 mM Tris pH 8.0, 0.1 M NaCl, 10 mM NaF, 1 mM Na_3_VO_4_, 0.5% NP-40, 10% Glycerol and 1 mM EDTA pH8.0) and continue to grind for 50–60 times. Cap the tube and incubate on ice for 10–15 min. Centrifuge at 12,000 rpm for 15 min. The supernatant was subject to western blot assay following standard protocols.

### Statistical analysis

The density of IB band was measured by Image J software. Statistical analysis was performed with GraphPad Prism software. The data shown in the Figures were representative of three or more independent experiments and were analyzed by one way student’s *t*-test, and *P* < 0.05 was considered statistically significant. Where exact *P* values are not shown, statistical significance is shown as with ns, not significant, *P < 0.05, **P < 0.01 and ***P < 0.001.

## Supplementary information

Supplemental information

reproducibility-checklist

## Data Availability

All relevant data are available from the corresponding author upon reasonable request.
