# Supplementary material for: Hippo signaling suppresses tumor cell metastasis via a Yki-Src42A positive feedback loop
Source: Cell Death Dis. 2021 Dec 3;12(12):1126. doi: 10.1038/s41419-021-04423-y (PMC8642408; doi:10.1038/s41419-021-04423-y)
Supplement: Supplementary file 1 — Supplemental information [file 41419_2021_4423_MOESM1_ESM.pdf]

**Supplemental information**

# Hippo signaling suppresses tumor cell metastasis via a Yki-Src42A positive feedback loop

**Ding et al.**

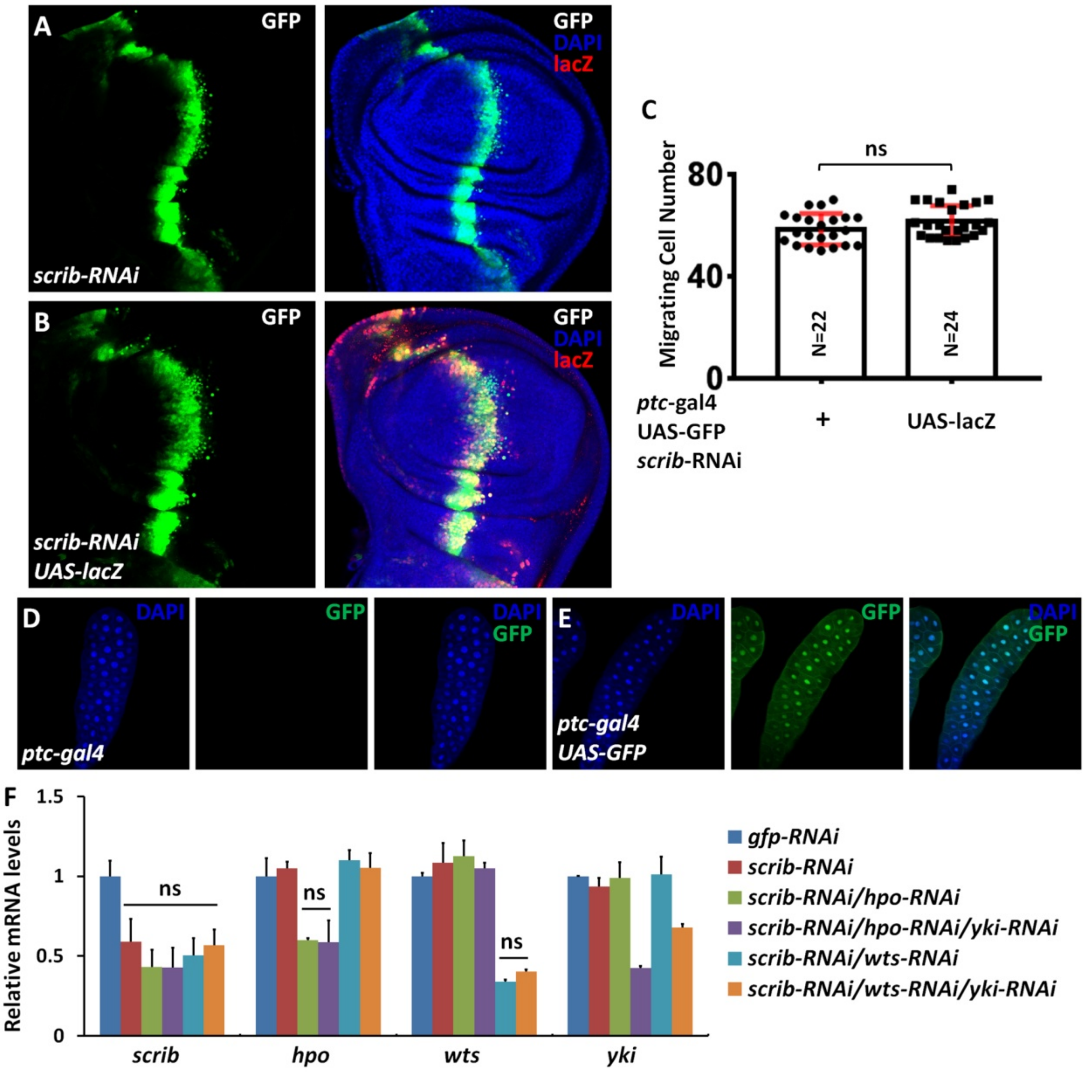


**Fig. S1** Removal the effect of Gal4 titration. (*A*) Knockdown of *scrib* triggered cell migration. (*B*) Co-expression lacZ did not affect *scrib*-RNAi-induced cell migration. (*C*) Quantification of migrating cell numbers in *A*-*B*. Data are presented as means ± SD of values. (*D*) A control salivary gland was stained with DAPI (blue) and GFP (green). (*E*) *ptc*-gal4 activated UAS-GFP expression throughout the salivary gland. (*F*) RT-qPCR showed the expression of indicated genes. Of note, different UAS numbers does not affect the efficiency of RNAi.

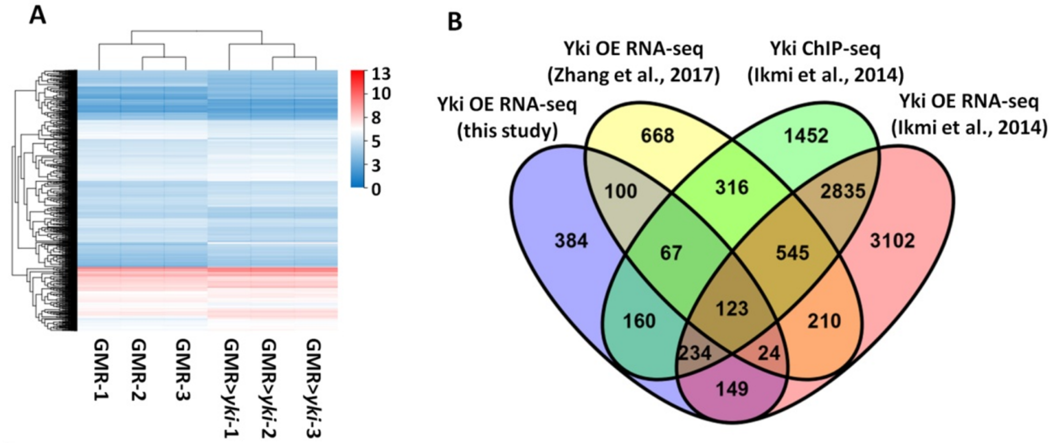


C

| | GMR | GMR>*yki* | fold change | Q value |
|---|---|---|---|---|
| *ex* | 647.1667 | 812.1933 | 1.32 | 0.00290 |
| *wg* | 90.3333 | 148.3333 | 1.72 | 0.00265 |
| *rho1* | 6861.3333 | 8172 | 1.25 | 0.000776 |
| *ds* | 774.6667 | 1044.3333 | 1.42 | 0.0000742 |
| *crb* | 1969.3333 | 2467.3333 | 1.32 | 0.00810 |
| *src42A* | 2905.3333 | 3523.3333 | 1.27 | 0.0000344 |

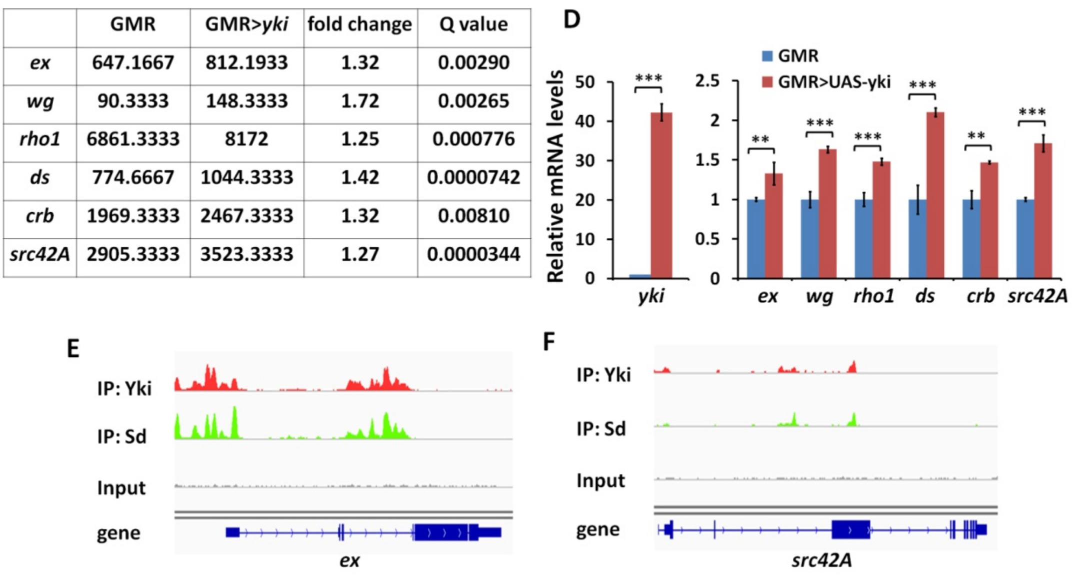


**Fig. S2** *src42A* is a potential target of Yki-Sd complex. (*A*) Heatmap of 1241 differentially expressed genes based on RNA-seq experiments from GMR heads (control) and GMR>*yki* heads. (*B*) Venn diagram showing the comparison between our RNA-seq data and those recently published in the literature. There were 123 genes overlapped in these four datasets. (*C*) The expression of indicated genes from GMR and GMR>*yki* samples. The numbers of expression represented fragments per kilobase of exon model per million mapped fragments (FPKM). (*D*) RT-qPCR showed the relative mRNA levels of indicated genes from GMR and GMR>*yki* samples. (*E*) ChIP-seq binding profiles (reads per million per base pair) for Yki and Sd at the *ex* locus in *Drosophila* eye discs. (*F*) Yki and Sd proteins occupied the *src42A* locus.

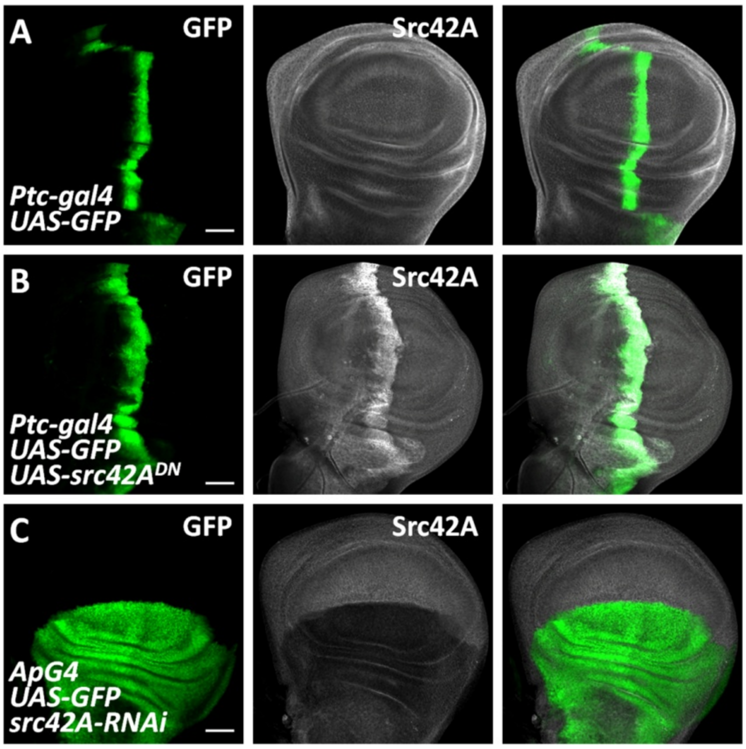


**Fig. S3** Validation of the Src42A antibody. (*A*) A control wing disc was stained to show GFP (green) and Src42A (white). Of note, *src42A* evenly expresses throughout the wing disc. (*B*) A wing disc expressing a dominant negative form of *src42A* (*src42A*$^{DN}$) was stained with GFP (green) and Src42A (white). Src42A antibody could recognize Src42A$^{DN}$ protein. (*C*) A wing disc expressing *src42A* RNAi by *ApG4* was stained with GFP (green) and Src42A (white). Upon *src42A* knockdown, the intensity of Src42A antibody staining decreased. Scale bars: 50μm for all images.

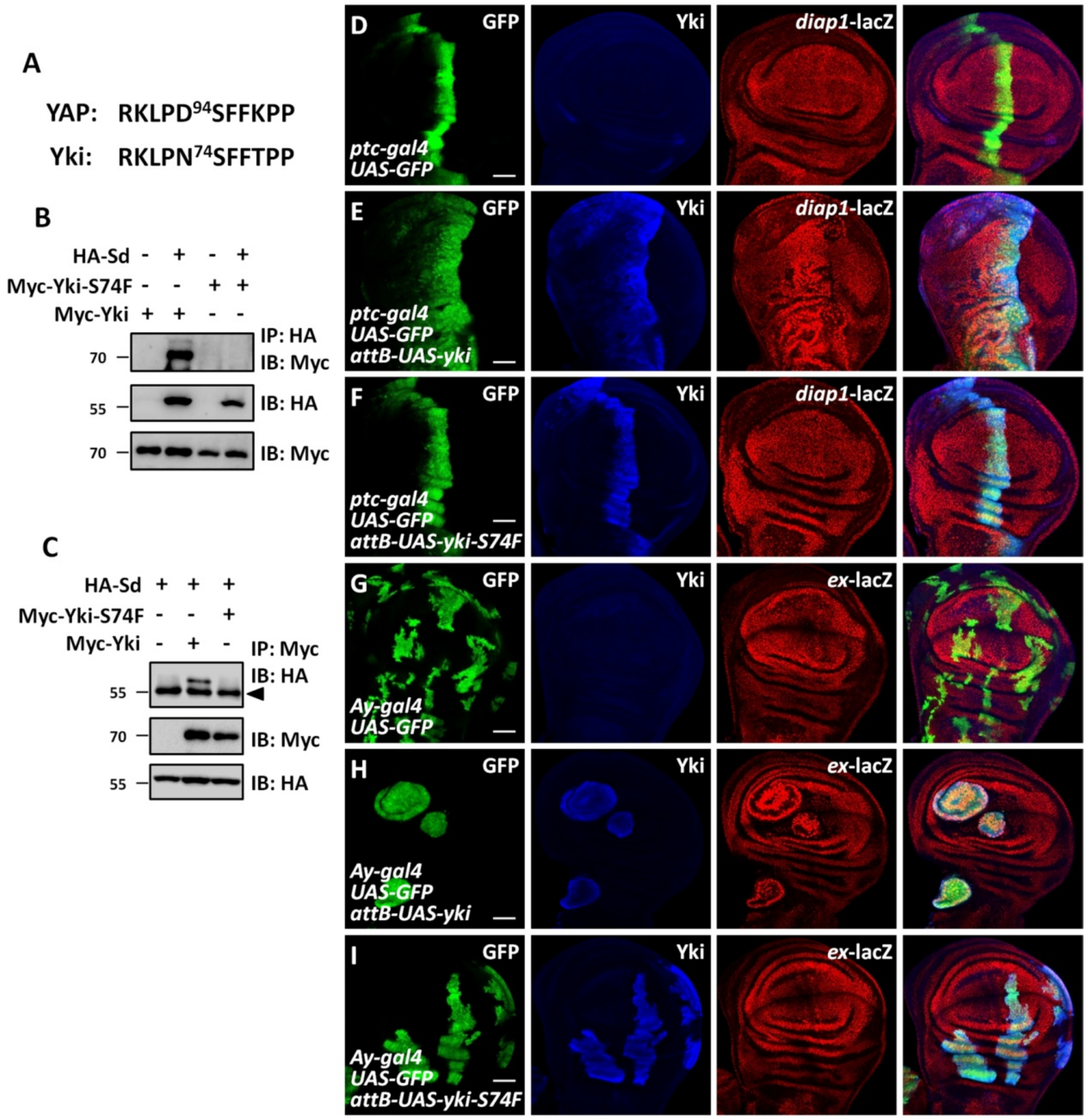


**Fig. S4** Yki-S74F fails to bind Sd and cannot activate canonical Yki-Sd target genes. (*A*) S74 of Yki protein was conserved with S94 of human YAP. (*B*) HA-Sd pulled down Myc-Yki, but failed to pull down Myc-Yki-S74F in S2 cells. (*C*) Myc-Yki-S74F did not associate with HA-Sd. The arrowhead marks the IgG bands. (*D*) A control wing disc was stained to show GFP (green), Yki (blue) and *diap1*-lacZ (red). GFP marks the expression pattern of *ptc-gal4* in the wing disc. (*E*) Overexpression of *yki* apparently increased *diap1*-lacZ. (*F*) Yki-S74F failed to activate *diap1*-lacZ expression. (*G*) A control wing disc expressing GFP by *Ay-gal4* was stained with GFP (green), Yki (blue) and *ex*-lacZ (red). (*H*) Overexpression of *yki* by *Ay-gal4* elevated *ex*-lacZ level. (*I*) Overexpression of *yki-S74F* did not affect *ex*-lacZ level. Scale bars: 50μm for all images.

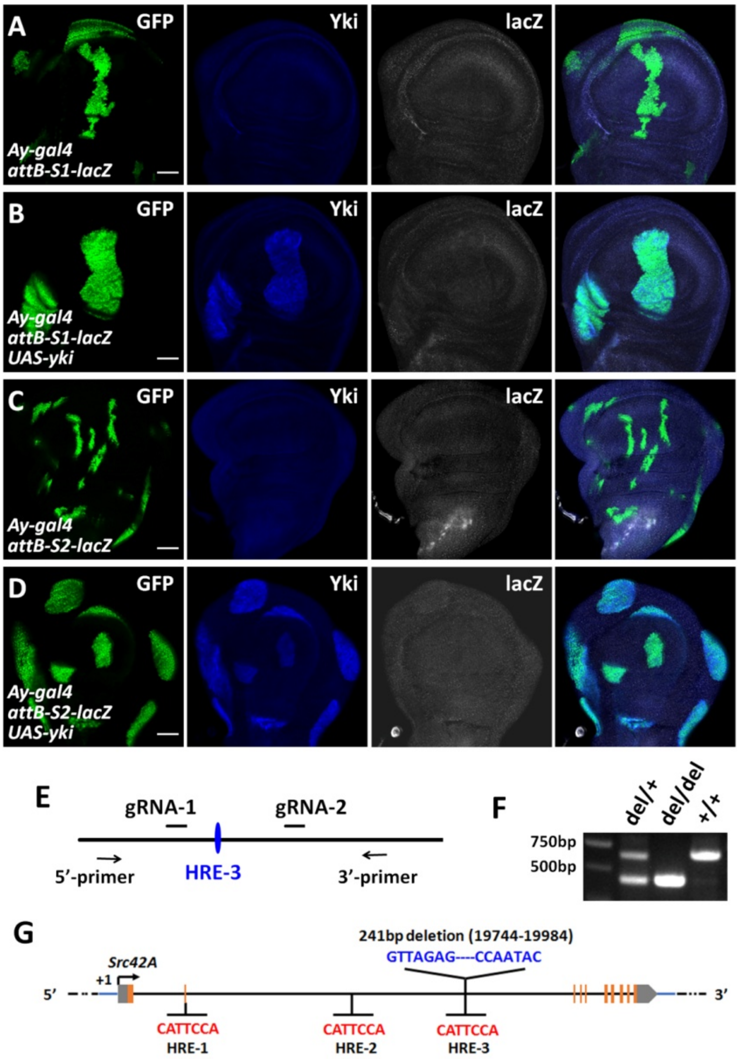


**Fig. S5** Yki activates *src42A* expression though its third HRE. (*A*) A control wing disc was stained to show the expression of GFP (green), Yki (blue) and S1-lacZ (white). S1-lacZ showed basal expression throughout the wing disc. (*B*) Overexpression of *yki* failed to increase S1-lacZ expression. (*C*) The expression pattern of S2-lacZ in the wing disc was shown. (*D*) Yki did not activate S2-lacZ expression. (*E*) Schematic view showing the third HRE (HRE-3) at the *src42A* locus. The gRNA-1 and gRNA-2 were designed for Cas9-mediated HRE-3 deletion. 5'-primer and 3'-primer were synthesized for subsequent PCR amplification. (*F*) The electrophoresis of PCR products. Deletion of the third HRE produced a truncated fragment. (*G*) Sanger sequencing showed that a 241bp (from 19744 to 19984) deletion, including the third HRE. Scale bars: 50µm for all images.

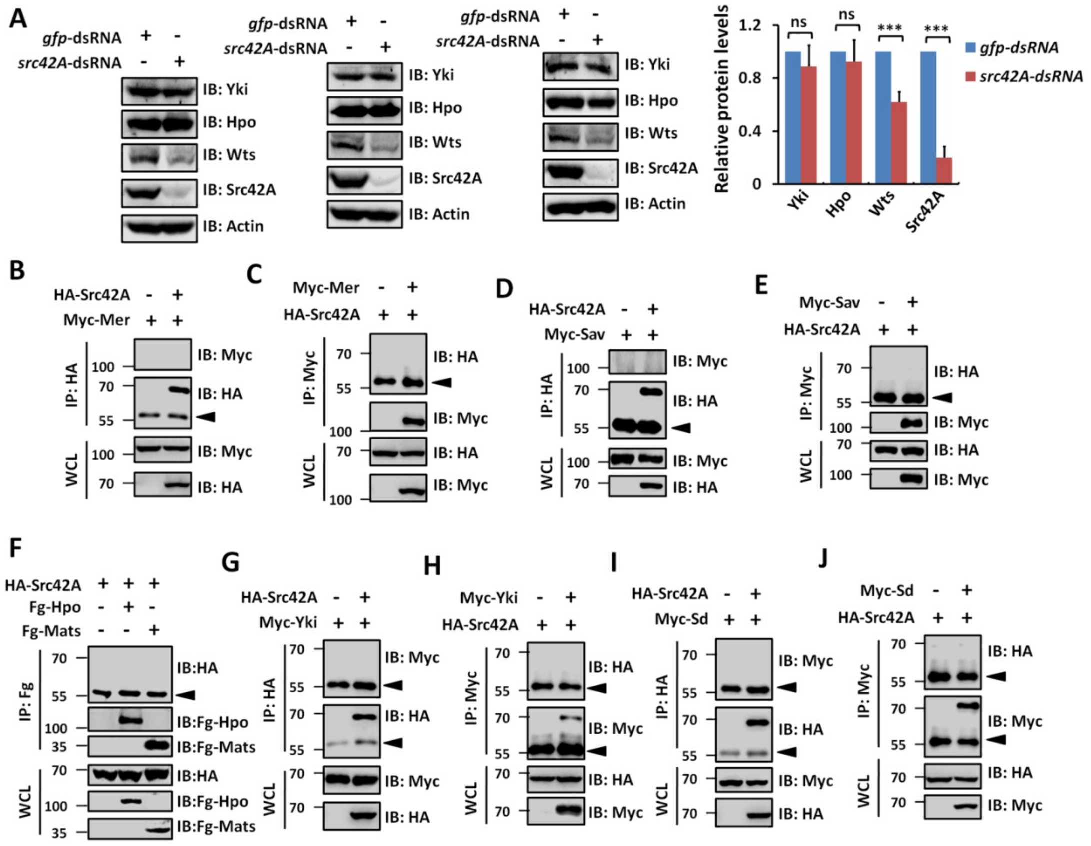


**Fig. S6** Src42A cannot interact with other components of the Hippo pathway. (*A*) Knockdown of *src42A* decreased Wts protein level. Quantification analyses were shown on right. (*B*) HA-Src42A did not pull down Myc-Mer. (*C*) Myc-Mer did not pull down HA-Src42A. (*D*) HA-Src42A did not pull down Myc-Sav. (*E*) Myc-Sav did not pull down HA-Src42A. (*F*) HA-Src42A could not interact with Fg-Hpo and Fg-Mats. (*G*) HA-Src42A did not pull down Myc-Yki. (*H*) Myc-Yki did not pull down HA-Src42A. (*I*) HA-Src42A did not pull down Myc-Sd. (*J*) Myc-Sd did not pull down HA-Src42A. For all above, arrowheads mark the IgG bands.

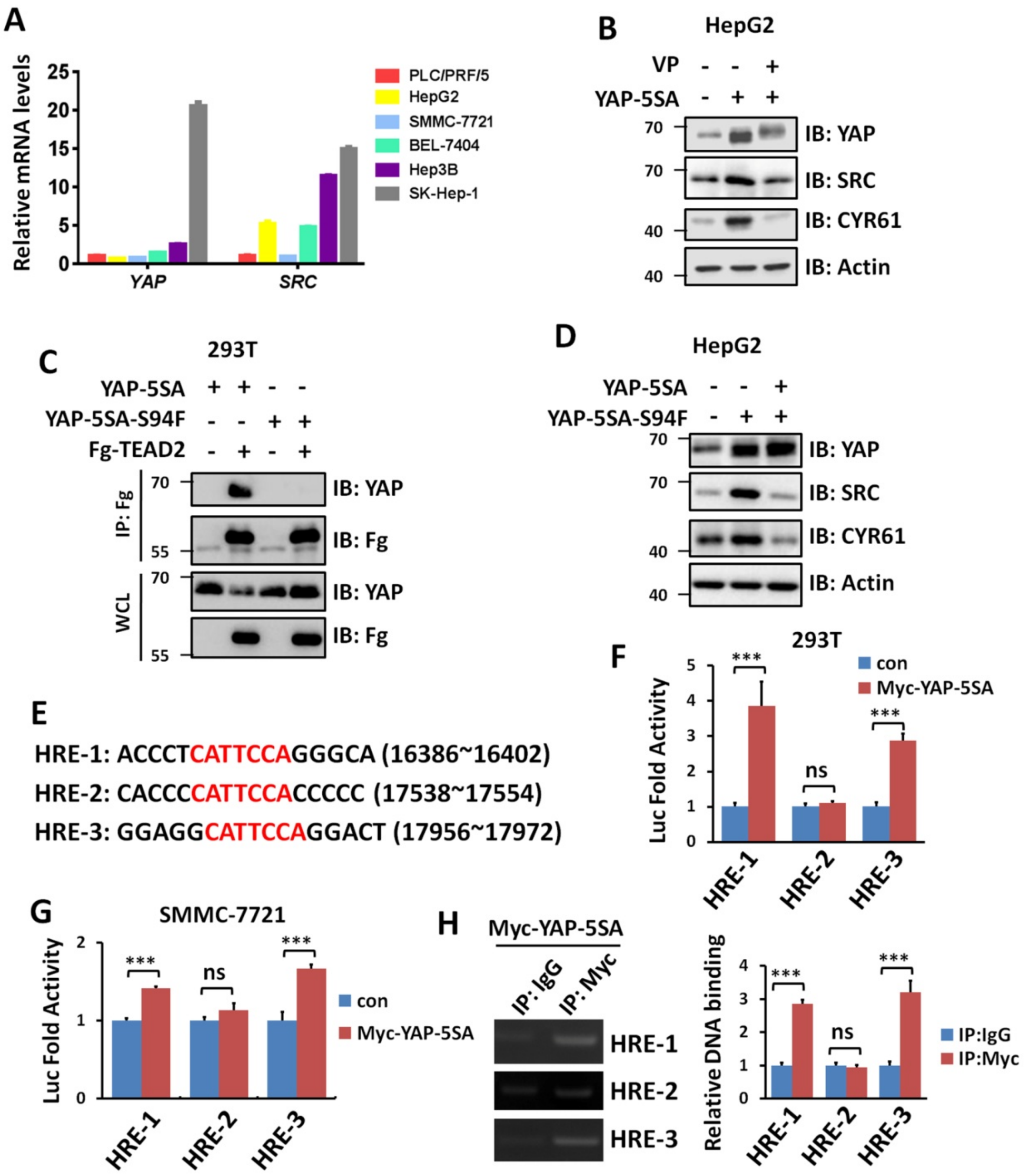


**Fig. S7** *SRC* is a direct transcription target of YAP-TEAD complex. (*A*) RT-qPCR showed the mRNA levels of *YAP* and *SRC* in different HCC cells. (*B*) YAP-5SA activated SRC expression, which was blocked by VP treatment. The well-known YAP target CYR61 acts as a positive control. Actin acts as a loading control. (*C*) YAP-5SA-S94F failed to interact with TEAD2. (*D*) YAP-5SA-S94F did not trigger SRC expression. CYR61 acts as a positive control, while Actin acts as a loading control. (*E*) *SRC* gene contained three TEAD binding sites in its first intron. (*F*) Luciferase assay showed the first and third HREs could respond to YAP-5SA in 293T cells. (*G*) Luciferase assay showed the first and third HREs could respond to YAP-5SA in SMMC-7721 cells. (*H*) ChIP-qPCR showed the interaction between

YAP-5SA with HREs in SMMC-7721 cells. Quantification analyses were shown on right. Of note, YAP-5SA is able to pull down HRE-1 and HRE-3.
